# Two Cysteines in Raf Kinase Inhibitor Protein Make Differential Contributions to Structural Dynamics In Vitro

**DOI:** 10.3390/molecules30020384

**Published:** 2025-01-17

**Authors:** Hyun Sang Cho, Mohammad Faysal Al Mazid, Eun-Young Lee, Md Abu Rayhan, Hyoun Sook Kim, Byung Il Lee, Hye Jin You

**Affiliations:** 1 Cancer Microenvironment Branch, Division of Cancer Biology, Research Institute, National Cancer Center, Goyang-si 10408, Republic of Korea; 76030@ncc.re.kr (H.S.C.); eylee@ncc.re.kr (E.-Y.L.); 2 Department of Cancer Biomedical Science, National Cancer Center Graduate School of Cancer Science and Policy, National Cancer Center, Goyang-si 10408, Republic of Korea; faysalharta16@gmail.com (M.F.A.M.); 99065@ncc.re.kr (M.A.R.); bilee@ncc.re.kr (B.I.L.); 3 Targeted Therapy Branch, Division of Precision Medicine, Research Institute, National Cancer Center, Goyang-si 10408, Republic of Korea; hskim@ncc.re.kr

**Keywords:** Raf kinase inhibitor protein, dimerization, cysteine, cancer

## Abstract

As a scaffolding protein, Raf kinase binding protein (RKIP) is involved in a variety of cellular pathways, including the Raf–MEK–ERK-cascade. It acts as a negative regulator by binding to its partners, making it an attractive target in the development of therapeutic strategies for cancer. Despite its structural stability as a monomer, RKIP may form a dimer, resulting in the switching of binding partners. It is still unclear how RKIP switches between monomeric and dimeric forms. Here, we identified the role of cysteine 133 in RKIP structural dynamics using recombinant human RKIP (rhRKIP) proteins purified from *Escherichia coli* BL21(DE3) cells. Mutation of alanine or serine instead of cysteine in RKIP proteins did not affect the biochemical characteristics, while dynamic light scattering and liquid chromatography (LC) quadrupole time-of-flight (Q-TOF) mass spectrometry (MS) suggested distinct peaks in solution, which were identified via LC–MS/MS analyses, and further clarified the role of cysteine in RKIP dimerization. rhRKIP dimer formation was abrogated by a 32-aa peptide mimicking the region between two RKIP proteins for dimerization. In addition, the 32-aa peptide and its short derivatives were investigated for effects on cancer cell viability. Taken together, our findings suggest that it may be possible to regulate RKIP function by controlling its dynamics with reducing agents, which could aid the targeting of cancer cells.

## 1. Introduction

The receptor tyrosine kinase (RTK)/RAS pathway is genetically altered in up to 47% of cancers, the highest rate among 10 canonical pathways examined in a previous study [1]. The altered RTK/RAS pathways in a variety of cancers drive tumor development and progression via dysregulated signaling cascades associated with proliferation and survival and are considered to be targets for the development of anticancer therapeutic strategies [2,3,4]. Increasing numbers of targeted drugs have been developed and applied in clinical treatment of cancer [2,3]. However, the development of resistance to these therapies remains an obstacle [5,6,7]. Resistance to targeted therapies may have genetic or nongenetic mechanisms [8]. Therefore, therapeutic strategies have been developed with combinations of drugs to achieve better responses based on differences in resistance mechanisms [8,9]. Acquired resistance may be explained by gain-of-function mutations upstream or downstream of the target or by activation of parallel signaling pathways [3,5,6,7,8,10]. For example, strategies targeting EGFR and survivin, an inhibitor of apoptosis, have been suggested to affect the RTK pathway as well as parallel pathways [11].

Raf kinase inhibitor protein (RKIP), also known as HCNP precursor protein or phosphatidylethanolamine-binding protein 1 (PEBP1), is a binding partner of Raf kinase 1 (Raf-1) [12], and interacts with Raf-1, MEK1, and ERK but not Ras. As a suppressor of tumor and metastasis [13], low RKIP expression is associated with poor prognosis in colon cancer [14,15], gastric cardia adenocarcinoma [16], gastrointestinal stromal tumor [17], and non-small-cell lung cancer [18]. Epigenetic modifications are associated with reduced RKIP expression in some cancers [19], supporting the association between RKIP expression and prognosis.

However, RKIP has pleotropic roles in a variety of signaling pathways through complex protein–protein interactions, which may not be possible to understand based only on altered expression levels [20,21,22]. For example, it appears to interact directly with microtubule-associated protein 1A/1B-light chain 3 (LC3), modulating autophagy under starvation, which is important for cellular metabolism [23], and with TANK-binding kinase 1 (TBK1), which is involved in inflammatory signaling [23]. With regard to cell plasticity [24], it interacts indirectly with Bach1 and affects its expression level. It is still unclear how RKIP interacts with various proteins in a situation-specific manner.

Structural flexibility may explain how RKIP modulates interactions with proteins [25,26,27,28]. Deiss et al. showed that RKIP undergoes dimer formation and thus switches its binding partner from Raf-1 to G-protein-coupled receptor kinase 2 (GRK2) in a serine 153 (Ser153)-phosphorylation-dependent manner [27], which was further found to play a role in β-adrenoceptor signaling [29]. Other studies have shown that the intramolecular salt bridge within the binding pocket of RKIP protein is important for binding to ligands, such as Raf-1 [28], and is affected by phosphorylation at Ser153 resulting in ligand switching [26]. Structural dynamics modulated in a phosphorylation-dependent manner seem to be important for RKIP function in a variety of cellular signaling pathways. However, it remains unclear how the structural dynamics of RKIP protein may be applied to lead to antitumorigenic signaling in cancer cells.

Cysteine as a free amino acid has roles in a variety of cellular responses depending on the redox environment [30,31,32]. Despite its reactivity and conservation through evolution, it plays these roles only when exposed on the surfaces of proteins [32]. It can undergo one-electron oxidation to radical species or two-electron oxidation leading to the formation of disulfide bonds or oxidized cysteines, such as cysteine sulfenic acid, cysteine sulfinic acid, or cysteine sulfonic acid, under specific conditions [31]. Its intra- and intermolecular interactions are considered to play significant roles in regulating the structures and dynamics of proteins [30]. However, how cysteine in RKIP protein contributes to structural dynamics, including dimerization, and whether it can be modulated remain unclear.

In this study, the roles of cysteine in RKIP protein were elucidated in vitro, and the protein was found to form a homodimer sensitive to reducing agents, such as dithiothreitol (DTT) and β-mercaptoethanol. We further identified a redox-sensitive region involved in RKIP homodimer formation by analyzing various site-specific mutant recombinant human (rh) RKIP proteins. These findings may be beneficial for modulating RKIP function and responses in targeted therapies associated with RTK/RAS pathways.

## 2. Results

### 2.1. Preparation of Recombinant Human RKIP Proteins for In Vitro Study

**Figure 1:**
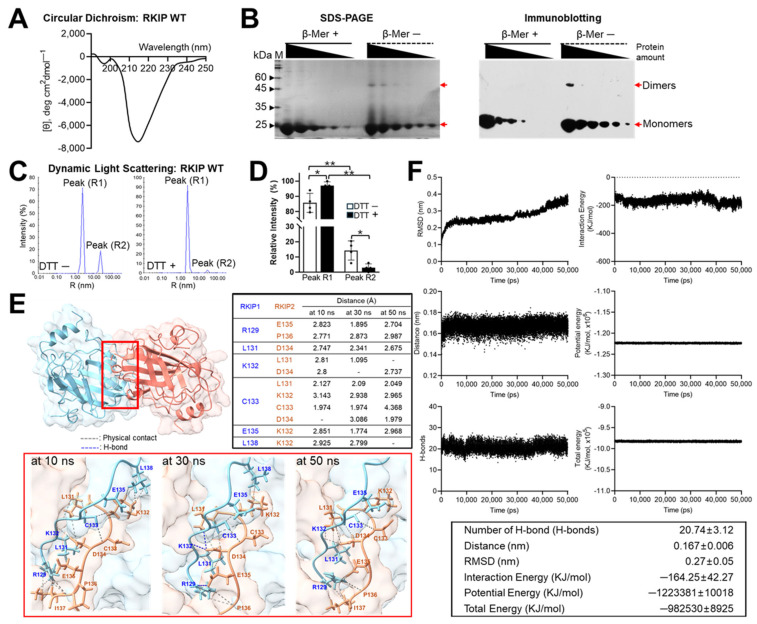
Preparation of recombinant human (rh) RKIP proteins and structural dynamics depending on reducing agents. (**A**) Purified rhRKIP protein samples obtained by affinity chromatography were analyzed by circular dichroism to examine their secondary structure. (**B**) rhRKIP proteins were prepared with sample loading buffer, with or without β-mercaptoethanol, and separated by SDS-PAGE. Gels were stained directly with Coomassie Brilliant Blue (**left**) or analyzed by immunoblotting using an anti-His antibody (**right**). (**C**) rhRKIP proteins were analyzed by dynamic light scattering (DLS) for molecular weight and homogeneity. Wild-type (WT) rhRKIP proteins showed two peaks (R1 and R2) corresponding to the molecular weights of the monomer (R1) and dimer or higher-order structures (R2) (**left**), with peak intensities affected by the presence of DTT (**right** graph). (**D**) Relative peak intensities for each measurement are expressed as the mean ± SE from at least three independent experiments. Statistical significance was assessed using the paired Student’s *t* test (* *p* < 0.05, ** *p* < 0.0001). (**E**) The predicted model structure of the RKIP dimer was visualized using UCSF ChimeraX (version 1.9) (**top**). The dimer interface (highlighted in the red box) was magnified to display detailed molecular interactions at 10 ns, 30 ns, and 50 ns during the 50 ns simulation (**bottom**). The distances between amino acids at the interfaces were measured at each time point (highlighted in the black box). Gray dotted line for physical contact (closest pair of atoms, predicted to contact physically); blue dotted line for H-bond. (**F**) The dynamics of the rhRKIP dimer model were calculated every 2 ps over a 50 ns simulation using the GROMACS platform (version 2024.0), three times. The analysis included RMSD, distance, number of hydrogen bonds (H-bonds), interaction energy, potential energy, and total energy. The root mean square deviation (RMSD) was used to assess differences in conformation of the protein dimer models. The distance between molecules within the predicted RKIP dimers was measured in angstroms (Å). Number of H-bonds was analyzed, and interaction energy was determined as the sum of van der Waals and Coulomb energies of the predicted RKIP dimers. The data presented represent the average values of three replicas and reflect the most consistent results obtained from at least three independent simulations using different dimer models.

To obtain rhRKIP proteins for in vitro experiments, transformed *Escherichia coli* BL21(DE3) cells containing the plasmid pET28B+ encoding histidine-tagged human RKIP cDNA (GenBank Accession: NM_002567) were utilized. In the presence of isopropyl β-D-1-thiogalactopyranoside, histidine-tagged rhRKIP proteins were successfully induced and appeared to be soluble after sonication (Supplementary Figure S1). By scaling up the culture, rhRKIP proteins were obtained and purified via affinity column chromatography using His–Ni-NTA (Supplementary Figure S1). The secondary structures of rhRKIP proteins were determined by circular dichroism analysis (Figure 1A), and found to be similar to recombinant RKIP proteins from other organisms [33,34].

### 2.2. Reducing Agent-Dependent Dimerization of rhRKIP

High-molecular-weight rhRKIP proteins (~45 kDa) appeared in the absence of the reducing agent β-mercaptoethanol, while a single band of only 22 kDa was observed in its presence (Figure 1B). These observations suggest a link between the dimerization process and the ability of the cysteine to form disulfide bonds. Labeling with anti-His antibody targeting His-tagged rhRKIP proteins confirmed the specific detection of RKIP dimers. Dynamic light scattering (DLS) analysis revealed two peaks with different molecular weights depending on the presence or absence of DTT (Figure 1C,D), supporting the suggestion of reducing agent-sensitive RKIP dimerization in vitro.

### 2.3. Computational Prediction of the Structure of Human RKIP Dimer

By simulating the dimer structure (Figure 1E,F and Supplementary Figure S2), we investigated whether RKIP forms stable dimers and identified potentially critical amino acids. For this purpose, we modeled the RKIP dimer structure using HADDOCK (version 2.4) [35] and the web server platform HADDOCK2.4, with input from the UniProt database (https://www.uniprot.org/, 3 June 2024) [36], generating several predicted dimer structures. Among these, three structures were processed to generate Protein Data Bank (PDB) files of the predicted dimer structures using ColabDock (https://neurosnap.ai/service/ColabDock, 31 July 2024) [37]. The representative dimer structure (Figure 1E) was visualized with UCSF ChimeraX (version 1.9) [38], and the PDB file thus generated was applied to GROMACS [39] to simulate the molecular dynamics of the predicted RKIP dimer structure according to the GROMACS-based simulation (force field: AMBER99SB-ILDN protein, nucleic AMBER94, 5894 atoms, a cubic simulation box (9.841 × 9.841 × 9.841) containing 29,835 water molecules (without ions)). We simulated molecular dynamics considering various parameters, including root mean square deviation (RMSD), to evaluate the stability of the RKIP dimers and measure conformational differences in the protein dimer models, the number of H-bonds indicating intramolecular interactions, the van der Waals energy, Coulomb energy, potential energy, and total energy. These parameters were calculated every 2 ps over a 50 ns simulation at least three times. Interestingly, the dimer model did not exhibit any marked dynamic fluctuations (Figure 1F and Supplementary Figure S2), suggesting that the predicted RKIP dimer structure remained stable up to 50 ns. In the detailed analysis of interactions within the RKIP dimer structure, the interface was magnified to display detailed molecular interactions at 10 ns, 30 ns, and 50 ns during the 50 ns simulation. The distances between amino acids, especially cysteine 133 (Cys133), but not cysteine 168 (Cys168), at the interfaces remained close at each time point. The model was further simulated up to 300 ns in a larger cube with more water one time, which supported the stable dimer structure (Supplementary Figure S2).

**Figure 2:**
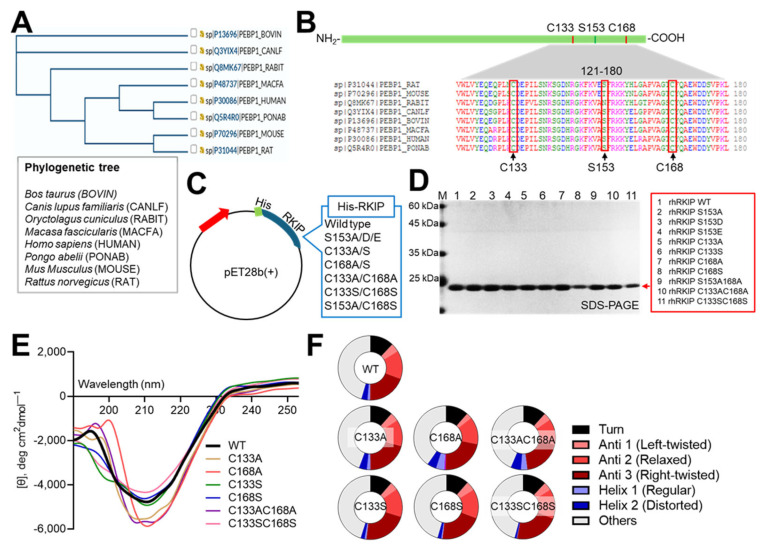
Phylogenetic tree and conservation of RKIP primary structure, and generation and circular dichroism characterization of rhRKIP mutant proteins. (**A**,**B**) Amino acid sequences of RKIP (PEBP1, UniProt ID: P30086) from various species were retrieved from the UniProt database (https://www.uniprot.org/, 3 June 2022) and subjected to alignment and phylogenetic analysis. (**B**) Amino acid residues 121–180 of RKIP from eight species were aligned, highlighting conserved cysteine residues at positions 133 and 168 and a serine residue at position 153 (arrowheads). (**C**) Schematic representations of plasmid constructs encoding mutant rhRKIP proteins. (**D**) Wild-type (WT) and mutant rhRKIP proteins were expressed, purified for in vitro studies, resolved by SDS-PAGE, and visualized using Coomassie Brilliant Blue. (**E**) Purified rhRKIP WT and mutant proteins were analyzed by circular dichroism to examine their secondary structures. The graph depicts the circular dichroism spectra of seven rhRKIP WT and mutant proteins: black solid line, WT; light brown line, C133A; red line, C168A; green line, C133S; blue line, C168S; violet line, C133A and C168A; pink line, C133S and C168S. (**F**) Based on the circular dichroism data, the secondary structures of the proteins were categorized into turns, various β-sheet types (left-twisted, relaxed, and right-twisted), and different α-helices (regular and distorted), along with other structural elements. The proportions of these structures within the rhRKIP proteins are represented as pie charts.

In phylogenetic analyses using the UniProt database [36,40], RKIP proteins in eight mammalian species (*Macaca fascicularis* [MF], *Pongo abelii* [PA], *Canis lupus familiaris* [CF], *Bos taurus* [BT], *Oryctolagus cuniculus* [OC], *Rattus norvegicus* [RN], *Mus musculus* [MM], and *Homo sapiens* [HS]) were classified into three groups, and human RKIP was more closely related to those of PA and MF than RN or MM (Figure 2A). Alignment of the eight mammalian RKIP amino acid sequences showed conservation of two cysteine residues in the C-terminus of the protein (Figure 2B), while Ser153 was conserved in six species (with the exceptions being RN and MM). Therefore, we generated several mutant RKIP clones via mutagenesis of Cys133 and Cys168 and Ser153, which were transformed into *E. coli* for in vitro protein preparation (Figure 2C,D and Supplementary Figure S1). Purified wild-type (WT) rhRKIP protein and six mutants in which two cysteines were substituted with alanine or serine were subjected to circular dichroism spectral analysis to determine whether cysteine substitution significantly affected the secondary structure of RKIP (Figure 2E,F). Based on the results, the rhRKIP WT and mutants exhibited similar characteristics in β-sheet structures (left-twisted, relaxed, and right-twisted), regardless of whether the cysteines were replaced with serine or alanine. However, differences were observed in the helix structures depending on the substituted amino acids. When one or both cysteines were replaced with alanine, both distorted and regular helix structures were more pronounced compared to WT RKIP protein. In contrast, helix structures were less prominent when cysteines were substituted with serine. UV and autofluorescence scanning did not show any significant differences between rhRKIP WT and mutant proteins.

### 2.4. Involvement of Cys133 in rhRKIP Homodimer Formation

**Figure 3:**
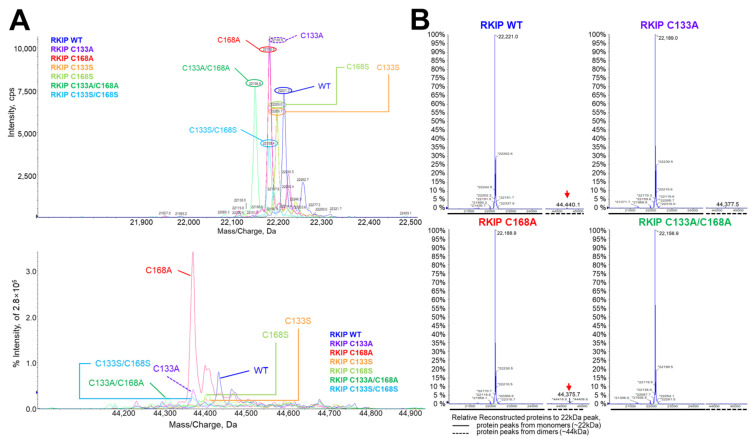
Validation of rhRKIP dimers by Q-TOF analysis based on cysteine at positions 133 and 168. Purified rhRKIP WT and mutant proteins with substitutions of Cys133 or Cys168 were subjected to Q-TOF analysis to detect high-molecular-weight RKIP dimers in addition to monomeric RKIPs. (**A**) Mass spectra of all rhRKIP WT and mutant proteins are shown in two regions: ~22 kDa (monomeric state, **top**) and ~44 kDa (high molecular weight dimer, **bottom**). The lines in the spectra are as follows: blue, WT; violet, C133A; red, C168A; orange, C133S; green, C168S; turquoise, C133A and C168A; sky blue, C133S and C168S. (**B**) The relative mass spectra of each rhRKIP protein are presented. The intensities of dimeric proteins relative to monomeric proteins are shown. Red arrows indicate high molecular weight proteins in the mass spectra, where detected.

**Figure 4:**
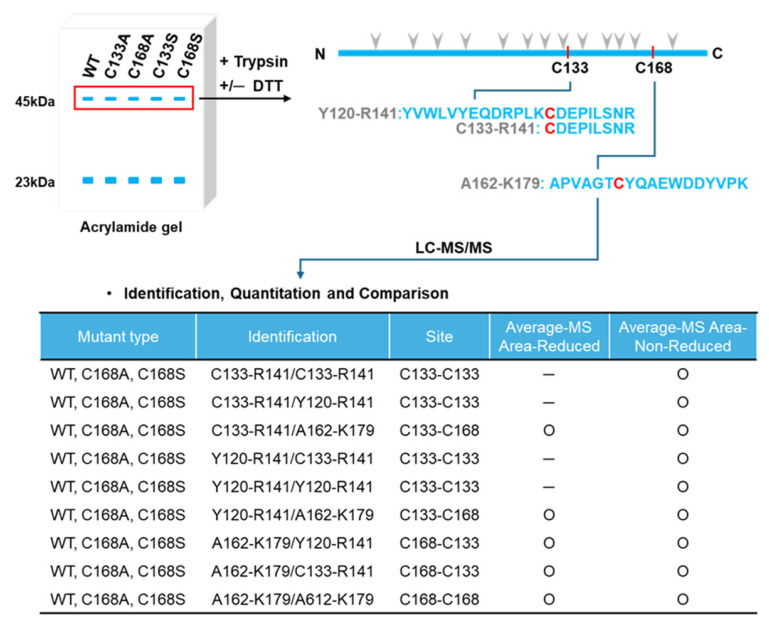
Identification of dimer-specific regions in the RKIP dimer in a cysteine-133-dependent manner using mass spectrometry. A simplified workflow for identifying dimer-specific regions (peptide sequences) in the presence or absence of DTT is shown (**top**). Purified rhRKIP WT and four mutant proteins were separated by electrophoresis, subjected to in-gel digestion with trypsin with or without DTT, and analyzed by mass spectrometry for peptide identification. With the exception of rhRKIP C133A and C133S mutants, rhRKIP proteins showed DTT-dependent mass peaks and peptides associated with cysteine at position 133 (**bottom**). Gray arrowheads: trypsin cleavage sites.

Next, we investigated whether rhRKIP WT and mutant proteins could form dimers in buffer at physiological pH (pH 7.4). To this end, we performed quadrupole time-of-flight (Q-TOF) analysis with rhRKIP WT and mutant proteins with substitution of cysteines with alanine or serine at positions 133 and 168 (Figure 3A,B). As expected, there were main peaks around 22 kDa for rhRKIP WT and mutant proteins, matching their molecular weights in monomeric form. The intensities of peaks around 44 kDa for rhRKIP WT and mutant proteins varied and were compared to those of the monomer peaks. Relative to the peaks around 22 kDa, those around 44 kDa were much weaker but appeared in rhRKIP WT and the C168A mutant, while they were absent in the C133A and C133A/C168A mutants, suggesting a role of cysteine at position 133 in rhRKIP dimerization. This was investigated further via liquid chromatography (LC)–mass spectrometry (MS)/MS analysis of protein samples in the presence or absence of the reducing agent DTT (Figure 4). Five rhRKIP WT and mutant proteins were cleaved by trypsin in the presence or absence of the reducing agent DTT, and the resulting peptides were analyzed via LC–MS/MS to identify differences based on the presence of DTT. Interestingly, the rhRKIP mutants C133A and C133S did not show any changes in peptide patterns upon treatment with DTT. However, the other proteins—rhRKIP WT, C168A, and C168S mutants—showed peptides associated with the reducing agent, specifically those linked to C133–C133, suggesting homodimer formation through cysteine at position 133.

### 2.5. Roles of Oligopeptides Including Cys133 and Its Neighbors in Homodimer Formation of rhRKIP

**Figure 5:**
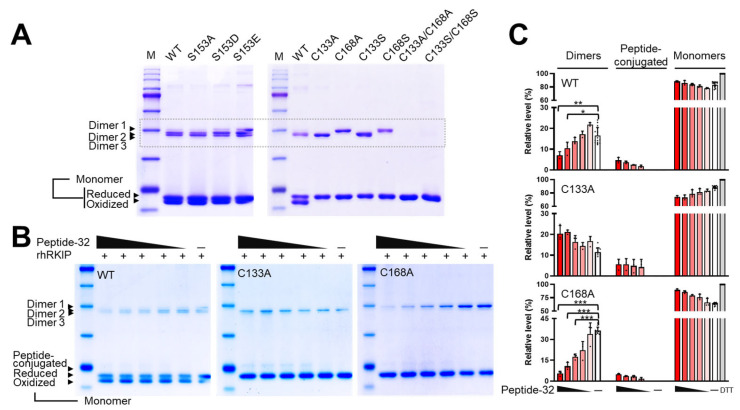
Inhibition of rhRKIP dimer formation by peptides mimicking the region near cysteine 133. Purified RKIP proteins were analyzed by electrophoresis in the absence of the reducing agent β-mercaptoethanol (**A**,**B**). (**A**) All rhRKIP WT and mutant proteins were separated by electrophoresis and stained with Coomassie Brilliant Blue to visualize protein bands. Data shown are representative results from at least three independent experiments. (**B**) Purified rhRKIP WT, C133A, and C168A proteins were incubated with peptide-32 for 30 min, then subjected to electrophoresis in the absence of reducing agents and visualized using Coomassie Brilliant Blue. Peptide-32 was added at varying molar ratios to the proteins (50:1, 25:1, 10:1, 5:1, 1:1). Data shown are representative results of at least three independent experiments. (**C**) Band intensities of dimers, monomers, and peptide-conjugated monomers (top of the monomer band region) were quantified using ImageJ software (version 1.54m). Relative intensities were calculated against the total protein (dimers, monomers, and peptide-conjugated monomers) and expressed as graphs using GraphPad Prism v.9 (GraphPad Software LLC, Boston, MA, USA). Data are presented as the mean ± SE from at least three independent experiments. Statistical significance was assessed using the paired Student’s *t* test. (* *p* < 0.05, ** *p* < 0.01, *** *p* < 0.0001).

LC-based analysis, Q-TOF, and LC–MS/MS indicated that RKIP formed homodimers, likely via a disulfide bridge between two RKIP protein monomers through C133–C133. Through electrophoresis, WT and mutant rhRKIP proteins were separated in the presence or absence of the reducing agent DTT (Figure 5 and Supplementary Figure S3). WT and all except two mutant rhRKIP proteins (C133A/C168A and C133S/C168S) formed dimers with different mobilities (faster: dimers 2 and 3; slower: dimer 1). WT and mutant rhRKIP proteins with substitution of Ser153 to alanine (A), glutamate (E), or aspartate (D) showed two monomers when separated in the absence of any reducing agents, while mutant rhRKIP proteins with substitution at Cys133 or Cys168 showed one monomer, suggesting that an intramolecular disulfide bridge or oxidized cysteine may affect mobility shifts on electrophoresis (Figure 5A). We further investigated whether peptides mimicking the region around Cys133 could disrupt homodimer formation depending on the presence of cysteine at this position. We designed a 32-amino-acid (aa) peptide containing a C133–C133 sequence (hereafter, peptide-32) and examined its effects on rhRKIP homodimer formation. WT and mutant rhRKIP proteins (1.5 μg) were incubated with peptide-32 at various molar ratios (1, 5, 10, 25, and 50 relative to rhRKIP protein) at 37 °C for 30 min and separated via electrophoresis (Figure 5B,C). We observed decreased dimer formation of rhRKIP WT and mutant C168A protein but not mutant C133A rhRKIP protein. As greater amounts of peptide-32 were added, WT and both mutant rhRKIP proteins showed more peptide-conjugated monomeric proteins, suggesting a negative role of peptide-32, specifically in dimer formation via Cys133.

**Figure 6:**
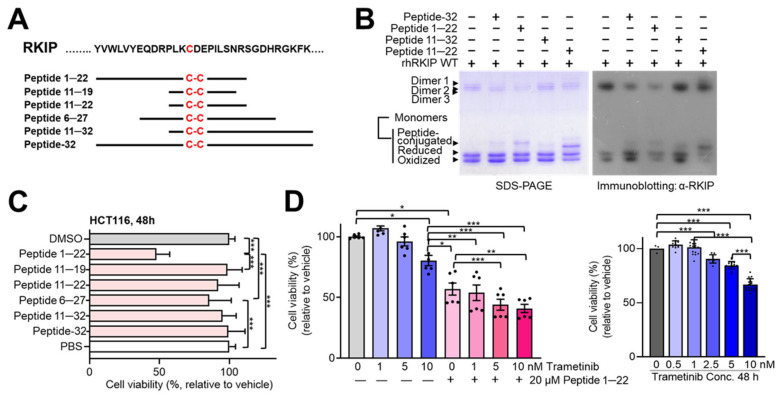
Peptides targeting the region near cysteine 133 may be beneficial for anticancer therapy. (**A**) Various peptides encompassing cysteine 133 and its neighboring regions were synthesized and analyzed. (**B**) rhRKIP WT proteins were incubated with various peptides at a molar ratio of 1:50 (rhRKIP:peptide) for 30 min at 37 °C in the absence of any reducing agent and subjected to SDS-PAGE. Gels were either stained directly with Coomassie Brilliant Blue (**left**) or analyzed by immunoblotting using an anti-RKIP antibody (**right**). (**C**) HCT116 cells (3 × 10^3^) were seeded in 96-well plates and incubated for 24 h, followed by treatment with peptides (20 μM) for 48 h. Cell viability was quantified by MTT assay. Peptide 1–22 was soluble only in DMSO, which served as a control. Relative cell viability compared to vehicle alone (PBS or DMSO) is presented as the mean ± SE from at least three independent experiments. Statistical significance was assessed using the paired Student’s *t* test. (**D**) HCT116 cells (3 × 10^3^) were seeded in 96-well plates and incubated for 24 h, followed by treatment with peptide 1–22 (20 μM), trametinib, or their combination for 48 h. Cell viability was quantified by MTT assay. Relative cell viability compared to the vehicle is presented as the mean ± SE from at least three independent experiments. Statistical significance was assessed using the paired Student’s *t* test. (* *p* < 0.05, ** *p* < 0.01, *** *p* < 0.0001).

Maintaining RKIP in the monomeric state may be beneficial by inhibiting the Raf–MEK–ERK cascade in certain types of cancer cells, potentially through disruption of protein–protein interactions rather than targeting enzyme active sites. To investigate this possibility, we designed additional peptides with core amino acid sequences of varying lengths (11–32 amino acids) (Figure 6). These peptides were tested for their ability to inhibit rhRKIP dimer formation via electrophoresis and immunoblotting and assessed for cytotoxicity using cell viability assays. Interestingly, peptide 1–22 inhibited rhRKIP dimer formation more effectively than peptide-32 and peptide 11–22, while peptide 11–32 showed no inhibitory effect (Figure 6B). These observations were confirmed via immunoblotting with anti-RKIP antibody. To examine cytotoxicity, we used the human colon cancer cell line HCT116 as a model (Figure 6C,D). This cell line harbors an oncogenic KRAS mutation at codon 13 (glycine to aspartate) but has no other mutations in genes associated with the RTK/RAS signaling pathway. When HCT116 cells were treated with synthesized peptides (20 μM) for 48 h, peptide 1–22 significantly reduced cell viability. Peptide 6–27 also reduced cell viability but to a lesser extent. Peptide 1–22 was further evaluated to determine whether it would be beneficial when combined with trametinib, an MEK1/2 inhibitor with anticancer activity [41,42]. Cell viability was reduced by trametinib alone, and this effect was further enhanced when combined with peptide 1–22. These observations suggest that peptide 1–22, which targets RKIP, may affect cancer cell viability both alone and combination with trametinib, particularly in cancer cells with an oncogenic mutation at Gly13 of Ras.

## 3. Discussion

We found that RKIP forms homodimers in solution via Cys133 in a manner dependent on the presence of reducing agents specific for disulfide bonds (Figure 1, Figure 3, and Figure 4). To inhibit RKIP dimerization and maintain its monomeric state, we designed peptides that mimic the C133–C133 interaction and its neighboring regions. These peptides effectively inhibited RKIP dimerization in vitro and demonstrated efficacy in reducing cell viability (Figure 5 and Figure 6).

Notably, we observed proteins with different mobilities during electrophoresis in the absence of a reducing agent. These results suggested that either intra- or intermolecular oxidation affects the binding of sodium dodecyl sulfate (SDS) due to steric hindrance, leading to differential mobility even for the same protein. In contrast, reducing agents linearized these proteins, resulting in consistent mobility and molecular weight (Figure 5). In addition, rhRKIP proteins with mutations at both Cys133 and Cys168 did not form dimers, while proteins with mutations at only one of these cysteine residues (either Cys133 or Cys168) still did, with differential dependence on reducing agents. Peptide-32 did not reduce HCT116 cell viability, which prompted us to design and examine the effects of shorter peptides (Figure 6). Among these, peptide 1–22 reduced cancer cell viability to a greater extent than the other peptides. Peptide 6–27 also slightly reduced cell viability, while peptide 11–32 as well as shorter peptides including peptide 11–22 and peptide 11–19 had no significant effect. These results suggested that the region between residues 120 and 133 may play a role in modulating RKIP dimerization, with corresponding residues in peptides potentially contributing to both RKIP dimerization and cell permeability. Further studies to investigate how these peptides function within cells are currently underway in our laboratory.

We directly demonstrated the involvement of cysteine at position 133 in RKIP dimer formation and that it can be targeted using peptides that mimic the interface between monomer units. However, rhRKIP proteins with single cysteine mutations formed distinct dimers depending on the specific cysteine residue: Cys133 contributed to the formation of the faster-migrating dimer (dimer 1), while Cys168 was associated with the slower-migrating dimers (dimers 2 and 3). In contrast, rhRKIP proteins in which both cysteines were substituted with alanine or serine showed complete loss of dimer formation. We are currently investigating the role of cysteines in RKIP function in cellular signaling.

Targeted therapies have been developed against numerous targets, including genes involved in the RTK/RAS pathways, such as EGFR, ERBBs, MET, RET, ALK, JAK2, and others. These genes transmit mitogenic signals to downstream effectors, including small G proteins, such as KRAS and HRAS, which activate the mitogen-activated protein kinase (MAPK) cascade (MAPKKK–MAPKK–MAPK), ultimately leading to cell cycle progression and proliferation [1,2,4]. To improve therapeutic outcomes, combination strategies to address mechanisms of resistance have been proposed [6,8,43,44]. In situ screening may be able to identify molecules for combating resistance involving the RTK pathway [43,44]. However, these approaches are still insufficient, as many patients exhibit a reduced response despite molecular diagnostics.

Here, we proposed a strategy based on protein dynamics, in which peptides mimicking regions critical for RKIP dimerization disrupt its dimer formation, thereby preserving its inhibitory function on mitogenic pathways that link MAPKKK to MAPKK (e.g., Raf kinase to MEKs). We focused on RKIP to enhance its inhibitory function by specifically targeting its dimerization. Notably, many other proteins form dimers in cells [45,46,47,48,49,50], which may play critical roles in therapeutic responses, including B-Raf [25,45,47,51,52]. Our approach could provide valuable insights for targeting the structural dynamics of other oncogenic proteins to facilitate the development of more effective therapeutic strategies.

## 4. Materials and Methods

### 4.1. Materials

Human RKIP cDNA (GenBank Accession: NM_002567) was obtained from DNASU Plasmid Repository (HsCD00674710, The Biodesign Institute/Arizona State University, Tempe, AZ, USA). Mouse monoclonal antibodies against His, RKIP, and horseradish-peroxidase-conjugated anti-mouse antibody were obtained from Santa Cruz Biotechnology, Inc. (Santa Cruz, CA, USA). Customized peptides were synthesized by Thermo Fisher Scientific Inc. (Waltham, MA, USA) and Peptron Inc. (Daejeon, Republic of Korea). HCT116 cell lines were obtained from the American Type Culture Collection (Manassas, VA, USA). Dulbecco’s modified Eagle’s medium (DMEM) and defined fetal bovine serum (FBS) were obtained from GIBCO (Grand Island, NY, USA). Trametinib (GSK1120212) was purchased from Selleck Chemicals LLC (Houston, TX, USA).

### 4.2. Sequence Alignment and Phylogenetic Analysis of RKIP in Mammals

A phylogenetic tree was generated using amino acid sequences of RKIP (PEBP1, UniProt ID: P30086) obtained from the UniProt database (https://www.uniprot.org/, 3 June 2022) [36,40]. We examined the conservation of two cysteine residues at positions 133 and 168 and a serine residue at position 153 across eight mammalian species: *M. fascicularis* (MF), *P. abelii* (PA), *C. lupus familiaris* (CF), *B. taurus* (BT), *O. cuniculus* (OC), *R. norvegicus* (RN), *M. musculus* (MM), and *H. sapiens* (HS).

### 4.3. Cloning of RKIP cDNA for Preparation of rhRKIP Wild-Type and Mutant Proteins

RKIP cDNA (GenBank Accession: NM_002567) was obtained from DNASU Plasmid Repository (HsCD00674710; The Biodesign Institute/Arizona State University, Tempe, AZ, USA) and cloned into the plasmid pET28B+ (Novagen, Merck KGaA, Darmstadt, Germany), using XhoI and NcoI for further application. Site-specific mutagenesis was performed to generate RKIP mutants by exchanging Cys133 or Cys168 with serine or alanine and Ser153 with alanine, glutamate, or aspartate using a Muta-Direct™ Site-Directed Mutagenesis Kit (#15071; iNtRON Biotechnology, Inc., Seongnam, Gyeonggi-do, Republic of Korea) according to the manufacturer’s protocol. Primers for site-specific mutagenesis were designed using Quick Change Primer design software (https://www.agilent.com/store/primerDesignProgram.jsp, 1 October 2021) (Supplementary Table S1). Point mutations at specific sites were confirmed by Sanger sequencing (Macrogen Inc., Seoul, Republic of Korea).

### 4.4. rhRKIP Protein Preparation In Vitro

#### 4.4.1. Transformation and Induction of Protein Expression by IPTG Treatment

For in vitro induction and purification of rhRKIP proteins, pET28B+ plasmids carrying RKIP cDNA with or without mutations were transformed into *E. coli* BL21(DE3). Colonies containing pET28B+ RKIP were grown in liquid culture overnight, and each cultured clone was diluted at a ratio of 1:1000 in LB media with kanamycin (50 μg/mL) at 37 °C, with shaking at 180 rpm until the optical density at 600 nm (OD_600_) reached 0.7. Then, IPTG was added to a concentration of 0.1 mM and incubated for 2–3 h at 37 °C with shaking. Cells were centrifuged at 4000× *g* for 20 min at 4 °C and used for protein extraction by resuspending pellets with lysis buffer (5 mM imidazole, 0.5 M NaCl, 20 mM Tris-HCl, pH 7.9), followed by sonication (Processor VC 505; Sonics & Materials, Inc., Newtown, CT, USA) in the presence of urea (3 or 6 M) or vehicle alone as a control. Sonication conditions were as follows: total process time, 15/30 s; pulse on time, 3 s; pulse off time, 30 s; and initial output level, 1.0. After sonication, samples were centrifuged at 12,000 rpm for 15 min to obtain supernatants, which were transferred to columns of Ni-NTA agarose (cat. # 20230; Sigma-Aldrich Corp. Merck KGaA, Darmstadt, Germany) for further purification.

#### 4.4.2. Purification of His-Tagged rhRKIP Proteins by Ni-NTA Chromatography

We added 5 mL Ni-NTA agarose resin to columns containing protein extracts and washed the sample with 10 column volumes of autoclaved double-distilled water (DDW). Next, rhRKIP proteins were bound to the resin by adding 10 column volumes of binding buffer (5 mM imidazole, 0.5 M NaCl, 20 mM Tris-HCl, pH 7.9), passed through the column at a very low flow rate. Then, the resin was washed with 20 column volumes of washing buffer (20 mM imidazole, 0.5 M NaCl, 20 mM Tris-HCl, pH 7.9) at a higher flow rate. To elute rhRKIP proteins, 20 mL elution buffer (300 mM imidazole, 0.5 M NaCl, 20 mM Tris-HCl, pH 7.9) was added and collected at a low flow rate. The eluted protein samples were pooled into several fractions with similar concentrations and dialyzed against PBS (135 mM NaCl, 2.7 mM KCl, 4.3 mM Na_2_HPO_4_, 1.4 mM KH_2_PO_4_, pH 7.4) by sealing with a dialysis membrane. For long-term storage, TBS–EDTA solution (200 mM Tris HCl, 1.5 M NaCl, 10 mM EDTA) was used for dialysis at 4 °C in a shaking incubator at 180 rpm (1 L TBS–EDTA solution per 1 mL eluted sample).

#### 4.4.3. Protein Quantification

Proteins purified via affinity chromatography were quantified using a bicinchoninic acid (BCA) assay for colorimetric detection with a Pierce BCA protein assay kit in accordance with the manufacturer’s instructions (Thermo Scientific, Rockford, IL, USA). Bovine serum albumin (BSA) was used to prepare a standard curve for quantification. The absorbance at 562 nm (A_562_) was measured using the VERSAMax microplate reader (Molecular Devices LLC., Sunnyvale, CA, USA).

### 4.5. Biochemical Characterization of rhRKIP Proteins

#### 4.5.1. Dynamic Light Scattering

Purified rhRKIP proteins were analyzed for molecular weight and homogeneity using dynamic light scattering (DLS) (Dynapro™; Wyatt Technology (BZ10123493), Waters Corp., Santa Barbara, CA, USA). Protein samples (100 μL) at various concentrations (100 μg/mL to 1.5 mg/mL) were loaded into a cuvette, placed in the machine, and measured. PBS–EDTA or TBS–EDTA was used as a negative control, while BSA was used as a positive control. Some protein solutions were measured in the presence or absence of 0.1 M DTT. At least three independent experiments were performed to obtain peak intensities for statistical analysis. Each experiment provided two peak intensities, from which relative peak intensity was calculated.

#### 4.5.2. Circular Dichroism Analysis

Purified proteins (50 μL at a concentration of 0.5 mg/mL) were subjected to circular dichroism analysis to characterize their secondary structure. Analysis was performed using a JASCO Circular Dichroism spectrometer (J-1500; Easton, MD, USA) at the Ochang Branch of the Korea Research Institute of Bioscience and Biotechnology (KRIBB) (Daejeon, Republic of Korea). Raw data were analyzed using BeStSel (Beta Structure Selection, https://bestsel.elte.hu/index.php, 4 March 2022) and plotted as circular graphs using GraphPad Prism v.9 (GraphPad Software LLC., Boston, MA, USA).

### 4.6. Mass Spectroscopy

#### 4.6.1. Quadrupole Time-of-Flight (Q-TOF) Analysis

To identify and quantify rhRKIP proteins in the monomeric and dimeric states, rhRKIP WT and mutant proteins were subjected to Q-TOF analysis, performed using Triple TOF 5600 (Thermo Scientific) by the National Cancer Center Proteomics Core Facility.

#### 4.6.2. LC–MS/MS Analysis

LC–MS/MS analysis was performed by the National Cancer Center Proteomics Core Facility [53]. Purified proteins (15 μg) were separated by polyacrylamide gel electrophoresis. Proteins with molecular weights of ~45 kDa were obtained by cutting out from the gels and dissolving in the presence or absence of DTT (100 mM), and subjected to in-solution tryptic digestion using a filter-assisted sample preparation protein digestion kit (Expedeon Inc., Encinitas, CA, USA) according to the manufacturer’s instructions. Peptides were extracted and cleaned up using C18 Zip Tips (Millipore, Bedford, MA, USA), followed by evaporation, and resuspended in 20 µL 0.1% formic acid for LC–MS analysis with a Q Exactive™ hybrid quadrupole Orbitrap mass spectrometer (Thermo Scientific) coupled with an Ultimate 3000 RSLCnano system (Thermo Scientific).

Database searching of all raw data files was performed in Proteome Discoverer 1.4 software (Thermo Scientific). MASCOT 2.3.2 and SEQUEST (https://proteomicsresource.washington.edu/protocols06/sequest.php, 29 November 2025) were used for database searching against UniProt [36,40]. Database searching against the corresponding reversed database was also performed to evaluate the false discovery rate of peptide identification [53]. The database searching parameters included up to two missed cleavages and allowed for full tryptic digestion, precursor ion mass tolerance of 10 ppm, fragment ion mass tolerance of 0.02 Da, fixed modification for carbamidomethyl cysteine, and variable modifications for methionine oxidation and N/Q deamination. We obtained a false discovery rate < 1% at the peptide level and filtered with high peptide confidence.

### 4.7. Polyacrylamide Gel Electrophoresis and Immunoblotting

#### 4.7.1. SDS-PAGE of Protein Samples With or Without Reducing Agents

Purified protein samples were mixed with sample loading buffer (2% glycerol, 2% SDS, 1% β-mercaptoethanol, 0.2% bromophenol blue, 12.5 mM Tris-HCl, pH 6.8) and heated at 95 °C for 7 min for electrophoresis [54]. Some protein samples were prepared without the reducing agent, β-mercaptoethanol, or boiling, to observe rhRKIP dimers. To examine the effects of peptides on rhRKIP dimer formation, protein samples were mixed with peptides for 30 min in the presence or absence of the reducing agent DTT, and then subjected to SDS-PAGE with sample loading buffer without reducing agent or boiling.

The protein samples were separated by 10–15% SDS-PAGE at 120 V for 2 h. The gels were stained with Coomassie Brilliant Blue solution for 25 min, followed by destaining with a 1:4:5 solution of acetic acid, methanol, and water. Then, they were imaged using a Geldoc IT^2^ 310 Imager (Ultra-Violet Products Ltd., Upland, CA, USA) with white light or a scanner.

#### 4.7.2. Immunoblotting

For immunoblotting, proteins were transferred from gels onto polyvinylidene difluoride membranes for 1 h at 100 V, which were blocked in Tris-buffered saline containing 0.01% Tween-20 (TBST) with 5% non-fat dried milk [54]. Then, they were incubated overnight with antibodies against His or RKIP in TBST with 2% non-fat dried milk at 4 °C, which were washed and incubated with horseradish-peroxidase-conjugated antibodies against mouse or rabbit as appropriate. Finally, the blots were developed with an enhanced chemiluminescence kit (West-ZOL Plus Western Blot Detection System; iNtRON Biotechnology) and quantification of band intensity on XAR-5 film (Eastman Kodak Co., Rochester, NY, USA).

### 4.8. Cell Culture and Viability Assay

The human colon carcinoma cancer cell line HCT116 was obtained from the American Type Culture Collection (Manassas, VA, USA) and maintained in DMEM/high glucose (SH30243.01; HyClone, Logan, UT, USA) with 10% FBS (10099141; Gibco) and 1× antimycotic–antibiotic (15240-062; HyClone) at 37 °C in a humidified 5% CO_2_ atmosphere [54]. In viability assays, HCT116 cells (3 × 10^3^ cells) were seeded on 96-well plates for 24 h, followed by treatment with peptides, trametinib, vehicle, or various combinations for an additional 48 h. Then, 3-(4,5-dimethylthiazol-2-yl)-2,5-diphenyltetrazolium bromide, a tetrazole (MTT), was added to each well during the last 3 h before harvesting, and the absorbance was measured according to the manufacturer’s instructions (Sigma-Aldrich Corp. Merck KGaA). Cell viability was calculated as the ratio of absorbance of treated cells to the absorbance of vehicle-treated cells.

### 4.9. Structural Modeling and Molecular Dynamics Simulation

The initial protein dimer structure was predicted using various software and web server platforms, including HADDOCK2.4 [35] and ColabDock (https://neurosnap.ai/service/ColabDock, 31 July 2024) [37], with protein sequences (P30089 by using the experimental model 2QYQ) obtained from the UniProt database (https://www.uniprot.org, 3 June 2024) [36,40]. To evaluate the stability of RKIP dimers, dimer structures were predicted using the HADDOCK2.4 platform [35]. Three models were selected for PDB generation using ColabDock [37] and visualized with UCSF ChimeraX (version 1.9) [38]. Molecular dynamics simulations of the dimer model were performed using GROMACS software (version 2024.0) [39], using the AMBER99SB-ILDN protein and nucleic AMBER94 force field with 5894 atoms in a cubic simulation box (9.841 × 9.841 × 9.841) containing 29,835 water molecules (without ions) as described in Supplementary Figure S2, following the procedure described in the online tutorial. Three models were simulated three times at 10 ns scale, and the best model was further simulated up to 50 ns scale three times. Based on these simulations, the RMSD was calculated to assess differences in conformation of the protein dimer models. In addition, intramolecular interactions were analyzed by calculating the number of H-bonds in the dimer model. Dimer stability was further evaluated from an energetic perspective by calculating van der Waals energy, Coulomb energy, potential energy, and total energy.

### 4.10. Statistical Analyses

All data are expressed as percentages of the control and are shown as means ± SE. Student’s *t* test was used to make statistical comparisons between groups. Values of *p* < 0.05 were considered significant.

## Data Availability

The original contributions presented in this study are included in the article/Supplementary Materials. Further inquiries can be directed to the corresponding author.

## Supplementary Materials

The following supporting information can be downloaded at: https://www.mdpi.com/article/10.3390/molecules30020384/s1, Figure S1: Preparation of recombinant human (rh) RKIP wild-type and mutant proteins; Figure S2: Structural models of hRKIP for molecular dynamics simulation; Figure S3: Purified rhRKIP WT and five mutant proteins showed differential motility during gel electrophoresis; Table S1: Primer information for site-directed mutagenesis.
