# Supplementary material for: Two Cysteines in Raf Kinase Inhibitor Protein Make Differential Contributions to Structural Dynamics In Vitro
Source: Molecules. 2025 Jan 17;30(2):384. doi: 10.3390/molecules30020384 (PMC11767649; doi:10.3390/molecules30020384)
Supplement: Supplementary file 1 [file molecules-30-00384-s001.zip › Supplementary Figures-PEBP1-dimers-01152025-final.pdf]

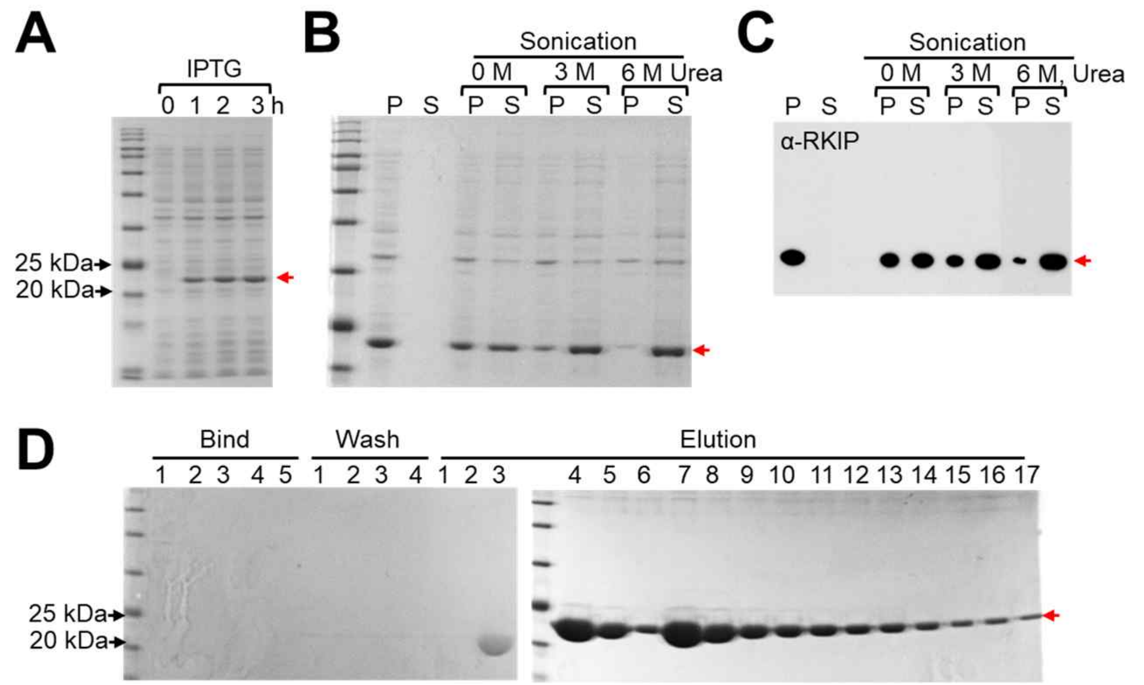

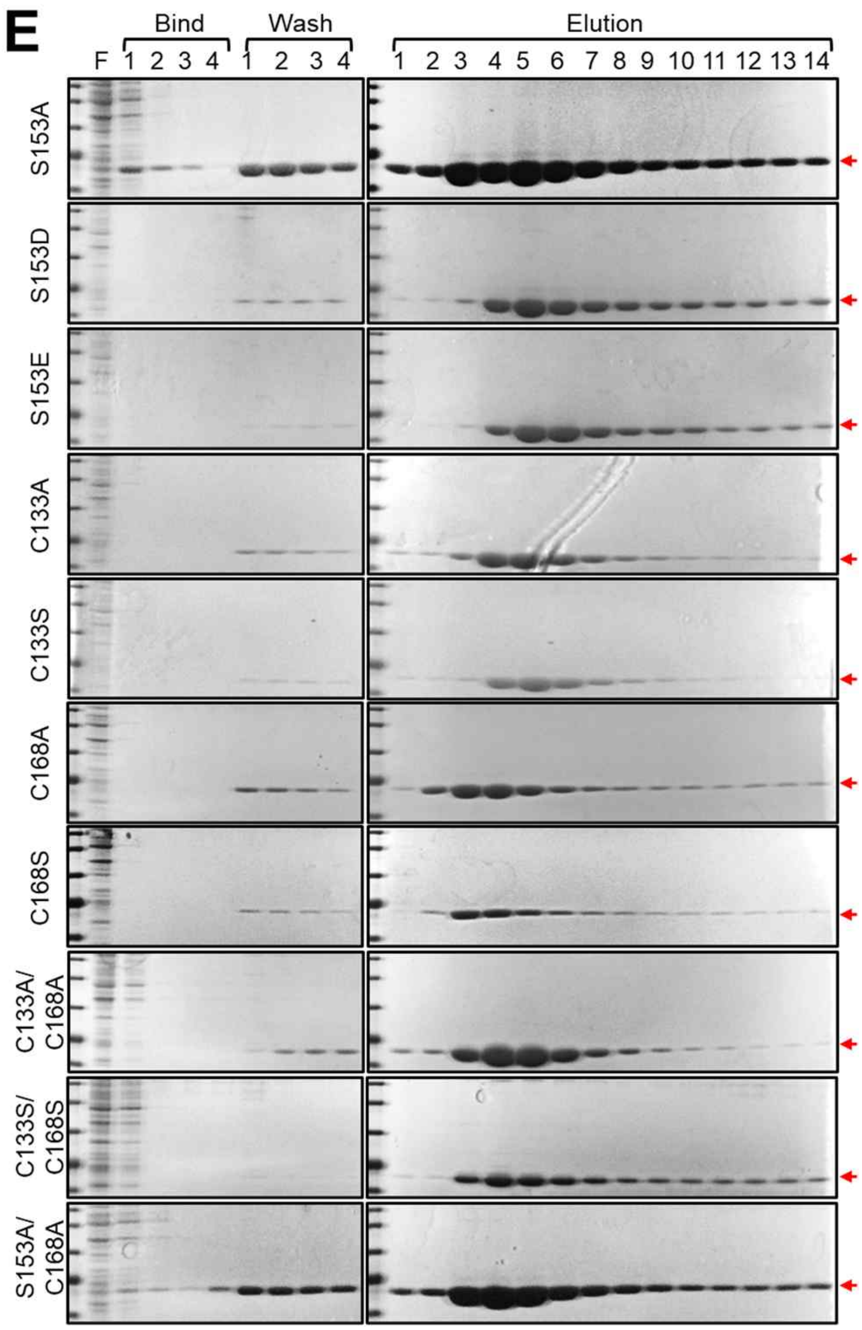

Supplementary Figure S1. Preparation of recombinant human (rh) RKIP wild type and mutant proteins. *BL21(DE3)* clone, transformed with a plasmid encoding human RKIP cDNA, was cultured and used for purifying rhRKIP proteins wild type and mutant proteins. (A) IPTG-induced protein expression in culture lysates was monitored by SDS-PAGE to optimize conditions for rhRKIP protein preparation. (B, C) Cultured samples were separated into pellets and supernatants by centrifugation to examine protein solubility. Gels were stained directly with Coomassie blue (B) or analyzed by immunoblotting using an anti-RKIP antibody (C). (D, E) Protein extracts from large-scale culture were purified by affinity chromatography. During the process, each step fraction was obtained and separated by SDS-PAGE followed by staining with Coomassie blue. (D) Fractions from rhRKIP wild type proteins preparation were shown. (D) Fractionated rhRKIP mutant proteins were shown. Data shown are the representative of at least two independent experiments.

A

Software and version
MD software: GROMACS 2024.0
Compilation Environment
Precision: Mixed precision
GPU Support: CUDA 12.0
SIMD Instructions: AVX2_256
Parallelization: OpenMP and MPI enabled
Force Field
Force Field Used
AMBER99SB-ILDN protein, nucleic AMBER94
GPU Support: CUDA 12.0
Water: TIP3P
Ions: AMBER ion parameters
Customization: No customization was applied
Simulation protocol
Energy Minimization
Algorithm: Steepest Descent
Maximum Steps: 50,000
Criteria: Maximum force < 10 kJ/mol/nm
NVT Simulation
Temperature 300 K
Temperature Coupling: V-rescale (τ = 0.1 ps)
Constant Volume: Applied
Total Time: 1 ns (500,000 steps)
NPT Simulation
Pressure 1.0 bar
Pressure Coupling: Parrinello-Rahman (τ = 5 ps)
Total Time: 1 ns (500,000 steps)
Isotropic Pressure Coupling: Applied
PME (Particle Mesh Ewald) for Electrostatics
PME Grid Spacing: 0.10 nm
Cutoff Distance: 1.2 nm (for Van der Waals and Coulomb interactions)
MD Simulation
Energy minimization was followed by NVT and NPT simulations sequentially.
Initial velocities were generated with a random seed
Initial velocities followed a Maxwell-Boltzmann distribution
Parallelization
8 CPU cores and 1 GPU (RTX 4080) were used for parallel processing.
The GPU was utilized for PME calculations.

B

PDB file	Model 1	Model 3	Model 4	Model1 (300 ns)
Total mass	42113.885 (5894 atoms)			
Force Field	AMBER99SB-ILDN protein, nucleic AMBER94			
Cube (nm)	9.841 x 9.841 x 9.841	9.817 x 9.817 x 9.817	9.805 x 9.805 x 9.805	10.645 x 10.645 x 10.645
Add water	29835	29506	29416	37700
Charge	0	0	0	0
Add ions	-	-	-	-
Temperature	300 ± 0.1K			
Pressure	1 ± 0.05 bar			

C

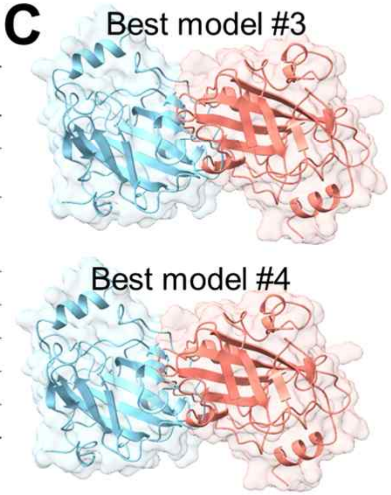

D

Contents	for Best model #3 for 10 ns	for Best model #4 for 10 ns	for Best model #1 for 300 ns
Number of H-bond (H-Bonds)	14.42±2.55	15.66±2.17	16.32±2.20
Distance (nm)	0.170±0.005	0.168±0.006	0.17±0.005
Interaction Energy (KJ/mol)	-123.54±25.80	-151.92±23.98	-160.19 ±38.93
RMSD (nm)	0.21±0.03	0.23±0.05	0.21±0.04
Potential Energy (KJ/mol)	-1212339±2736	-1213894±1467	-1536750±1635
Total Energy (KJ/mol)	-973589±3153	-975351±1793	-1236217±1975

E

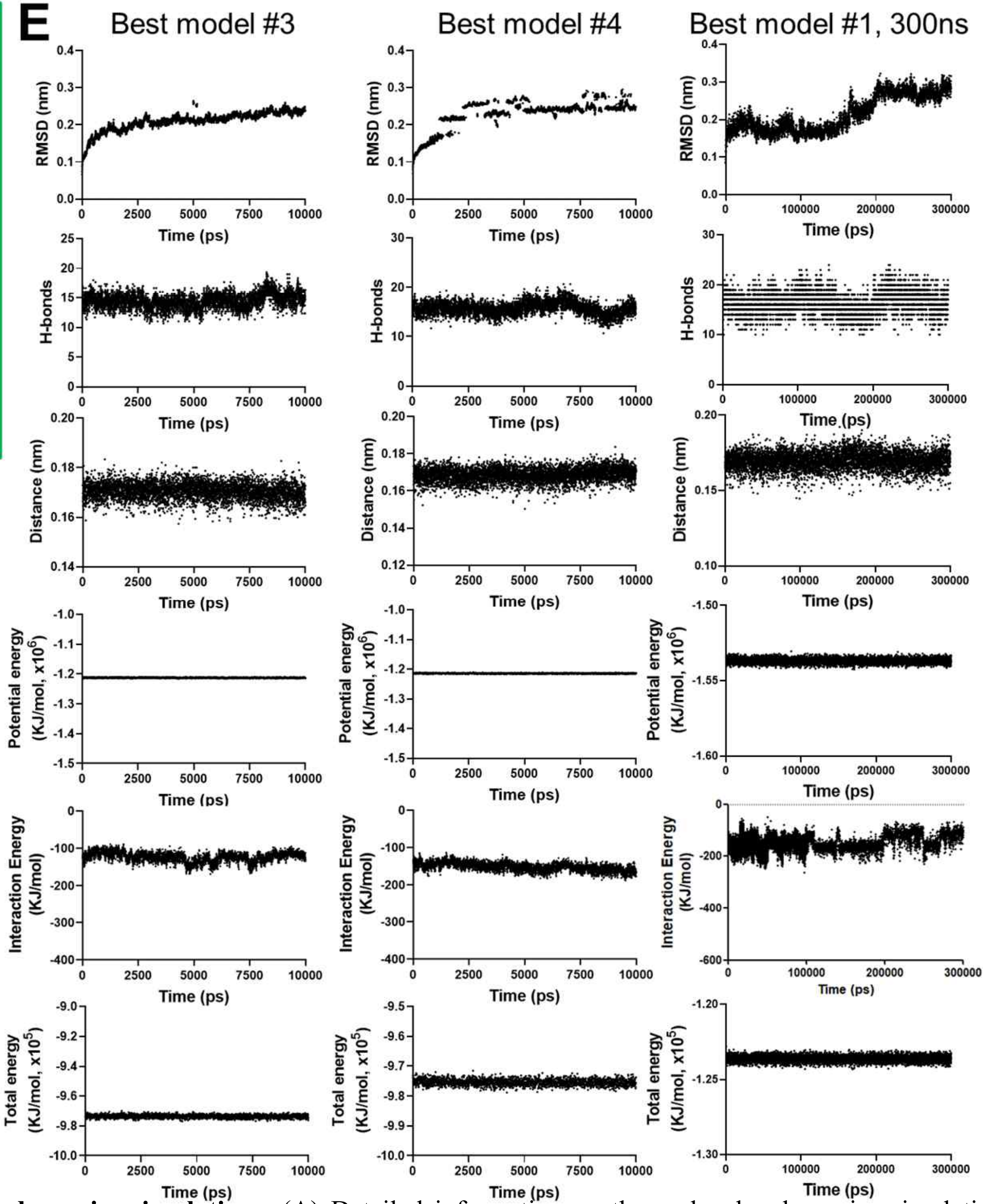

Supplementary Figure S2. Structural models of hRKIP for molecular dynamics simulation. (A) Detailed information on the molecular dynamics simulation using GROMACS. (B) Conditions for molecular dynamics simulations with three dimer models predicted by ColabDock. (C) The predicted RKIP dimer structure was visualized using UCSF ChimeraX. (D, E) The dynamics of the hRKIP dimer model were calculated every 2 ps over a 10 ns simulation for Model 3 and 4, or everty 50 ps over 300ns simulation for Model 1-300 ns using the GROMACS platform, repeated three times. The analysis included RMSD, distance, number of hydrogen bonds (H-bonds), interaction energy, potential energy, and total energy. The root mean square deviation (RMSD) was used to assess conformational differences in the protein dimer models. The distance between molecules within the predicted RKIP dimers was measured in angstroms (Å). The number of H-bonds was analyzed, and interaction energy was calculated as the sum of van der Waals and Coulomb energies of the predicted RKIP dimers. The data presented represent the average values of three replicas and reflect the most consistent results obtained from at least three independent simulations using different dimer models.

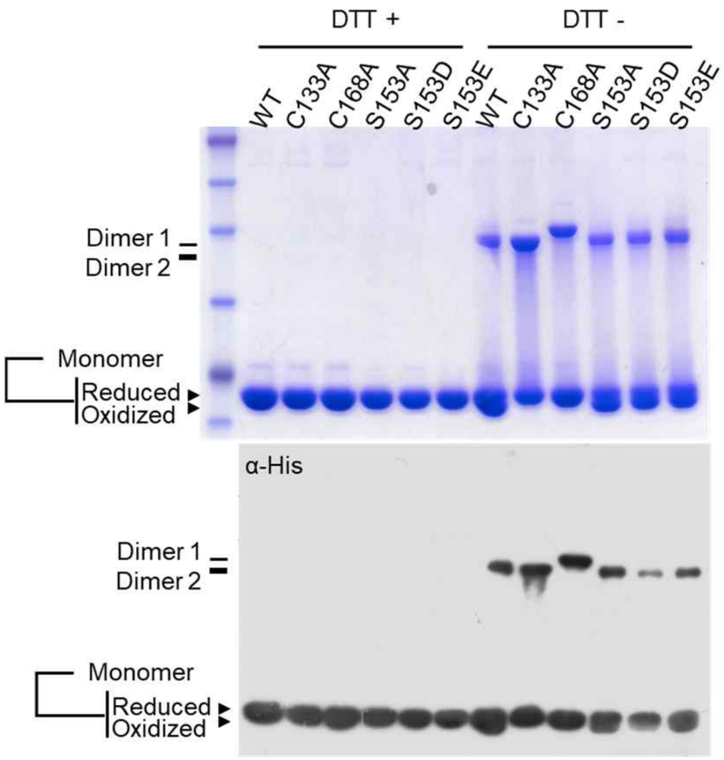

Supplementary Figure S3. Purified rhRKIP WT and five mutant proteins showed differential motility during gel electrophoresis. Puified proteins were separated by gel electrophoresis in the presence or absence of DTT, the reducing agent. Gels were stained directly with Coomassie blue (top) or analyzed by immunoblotting using an anti-His antibody (bottom). Data shown are the representative of at least three independent experiments.
